# Preparing for the KIL: Receptor Analysis of *Pseudomonas syringae* pv. *porri* Phages and Their Impact on Bacterial Virulence

**DOI:** 10.3390/ijms21082930

**Published:** 2020-04-22

**Authors:** Dominique Holtappels, Alison Kerremans, Yoni Busschots, Johan Van Vaerenbergh, Martine Maes, Rob Lavigne, Jeroen Wagemans

**Affiliations:** 1 Laboratory of Gene Technology, Department of Biosystems, KU Leuven, Kasteelpark Arenberg 21 box 2462, 3001 Heverlee, Belgium; dominique.holtappels@kuleuven.be (D.H.); alison.kerremans@kuleuven.be (A.K.); yoni.busschots@kuleuven.be (Y.B.); rob.lavigne@kuleuven.be (R.L.); 2 Department of Crop Protection, Institute for Agricultural and Fisheries Research, Burg. Van Gansberghelaan 96, 9820 Merelbeke, Belgium; johan.vanvaerenbergh@ilvo.vlaanderen.be; 3 Independent Researcher, Willem Tellstraat 20, 9000 Ghent, Belgium; mamaeswt20@hotmail.com

**Keywords:** phage biocontrol, phage receptors, bacterial virulence

## Abstract

The prevalence of *Pseudomonas syringae* pv. *porri* (Pspo) in Belgium continues to increase and sustainable treatments for this pathogen remain unavailable. A potentially attractive biocontrol strategy would be the application of bacteriophages. The ideal application strategy of phages in an agricultural setting remains unclear, especially in a field-based production such as for leek plants in Flanders. Therefore, more insight in bacteria–phage interaction is required, along with the evaluation of different application strategies. In this study, we further characterized the infection strategy of two Pspo phages, KIL3b and KIL5. We found that both phages recognize lipopolysaccharide (LPS) moieties on the surface of the bacterium. LPS is an important pathogenicity factor of Pspo. Our data also suggest that KIL5 requires an additional protein in the bacterial cytoplasmatic membrane to efficiently infect its host. Virulence tests showed that this protein also contributes to Pspo virulence. Furthermore, a cocktail of both phages was applied in a seed bioassay. A combination of KIL3b and KIL5 reduced the bacterial concentration 100-fold. However, in vitro Pspo resistance against phage infection developed quite rapidly. However, the impact of this phage resistance might be mitigated as is suggested by the fact that those resistance mutations preferably occur in genes involved in LPS metabolism, and that the virulence of those mutants is possibly reduced. Our data suggest that the phage cocktail has promising potential to lower the prevalence of Pspo and to be integrated in a pest management strategy. Targeted research is needed to further explore the applicability of the phages in combination with other disease control strategies.

## 1. Introduction

The application of bacteriophages as crop-protection agents dates back to 1924 when bacteriophages were first utilized in field trials against *Xanthomonas campestris* pv. *campestris* [1,2]. In recent years, the interest in the application of phages in a biocontrol setting is resurfacing as an alternative biocontrol strategy [3]. Indeed, the need for alternative treatment strategies is great in view of the restrictions on the use of antibiotics and copper-based chemicals, for environmental and public safety reasons [4,5]. Moreover, resistance towards these traditional compounds has been widely reported for different bacterial genera including *Erwinia*, *Pseudomonas,* and *Xanthomonas* [6]. As such, phage biocontrol is being considered as a potential strategy to tackle bacterial diseases in crop production, as it fits within the framework of integrated pest management [3,7].

One of the most prominent phytopathogenic bacteria is *Pseudomonas syringae*. This species is able to cause disease in a wide range of economically relevant crops such as tomato, kiwifruit, bean, etc. [8]. One of its pathovars is *Pseudomonas syringae* pv. *porri* (Pspo), the causal agent of bacterial blight in leek plants. This pathogen was first described by Lelliot et al. in 1952 in the United Kingdom [9]. Since then, it has become widespread and has been reported in Europe, Oceania, North America, and Asia [10]. Pspo typically causes leaf curling and leaf lesions in young leek plants while older plants develop water-soaked spots [11,12]. The main plant host is leek (*Allium porrum*), but also other members of the *Allium* family such as onions (*Allium cepa*) and shallots (*Allium cepa* var. *aggregatum*) can be infected [12]. In Belgium, we recently reported an increase in Pspo prevalence in leek plants [10]. The residing Pspo strain population was analyzed and two genotype groups were revealed based on BOX-PCR data. To develop a sustainable disease management strategy for Pspo in leek plants, we isolated and characterized five Pspo infecting phages, KIL1-5, and validated them in bioassays and field trials [13]. These bioassays showed that the phages are able to reproduce inside the plant tissue when co-injected with the bacterial host. However, in field trials, these phages showed only a minor capacity for disease suppression, as has also been reported for other pathosystems [2]. 

One of the limitations of phage applications is the narrow host range of most bacteriophages [14]. As such, the selection of a phage cocktail is mainly based on its ability to infect a wider diversity or range of host strains. The host range of a phage can be defined as the strains (or species) of a particular bacterium that are susceptible to the phage and facilitate a successful reproduction of this phage [15]. In other words, being successful at every stage of the infection, i.e., adsorption, injection of the genetic material, transcription and translation of the viral proteins, maturation of the phage particle, and finally the release of the phage progeny [14]. In the adsorption phase, the phage attaches to the bacterial surface by means of an interaction between the phage capsid proteins and their receptor site on the bacterial cell wall. In the case of Gram-negative bacteria, phages are known to recognize different structures on the outer membrane such as sugar moieties and membrane proteins [16]. Indeed, different sites on the outer surface of bacterial phytopathogens such as exopolysaccharides, colanic acid residues, lipopolysaccharides, and type IV pili are described as crucial for phage attachment and infection [17,18,19,20]. 

In this investigation, the phage infection strategy of two Pspo phages was studied. Moreover, the influence of phage resistance on bacterial virulence was evaluated by infecting adult leek plants with phage-resistant mutants. The overall performance of a phage cocktail containing KIL3b and KIL5 as an agent for priming of leek seeds was assessed, as was the resistance frequency. These mutants were analyzed by whole genome sequencing to determine the mutations that are responsible for phage resistance in the case of Pspo.

## 2. Results

### 2.1. Subsection Identification of Genes Involved in the Attachment of KIL3b and KIL5 to *Pseudomonas syringae* pv. *porri*

**Figure A1:**
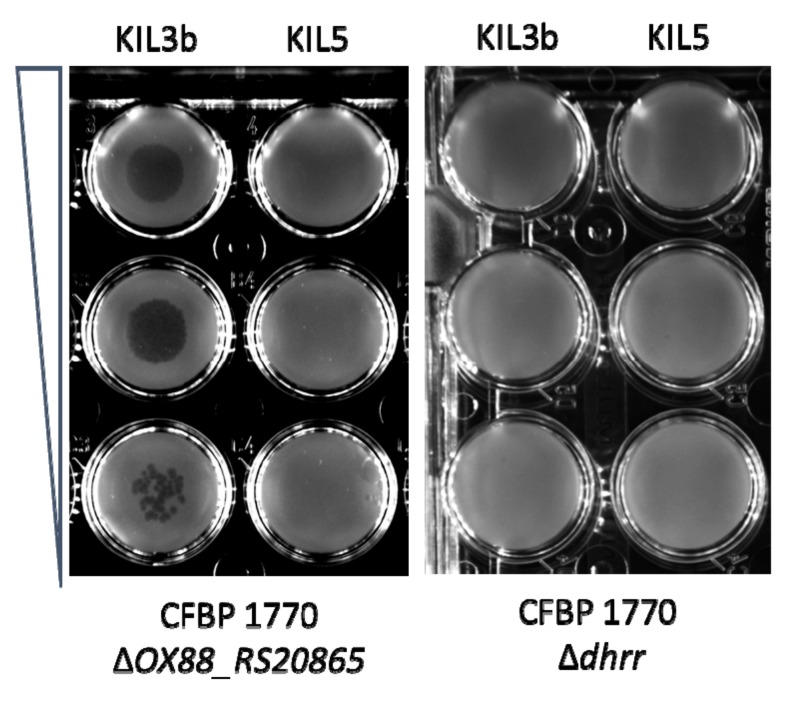
Spot assay showing the resistance and susceptibility of the transposon knock-out mutants to KIL3b and KIL5. On the left, CFBP 1770 Δ*OX88_RS20865* sensitive to KIL3b and resistant to KIL5, and on the right, CFBP 1770 Δ*dhrr* resistant to both phages. A dilution series (10^6^ – 10^5^ – 10^4^ PFU/mL) of phage was spotted on a bacterial lawn containing CFBP 1770 Δ*OX88_RS20865* and CFBP 1770 Δ*dhrr*.

A knock-out library of Pspo CFBP 1770 was created and resulted in 715,400 transformants covering the full genome of *Pseudomonas syringae* pv. *porri* [21]. After phage infection, up to 24 phage-resistant clones were picked and retested for phage resistance (data not shown) and also their cross-resistance to the other phage was tested. In total, DNA from up to seven different clones was isolated and the location of the transposon was determined using TAIL-PCR. The sequence flanking the transposon was analyzed using tBLASTx. An overview of the different genes found to be associated with the phage-resistant phenotype is provided in Appendix Table A1. In summary, KIL3b- and KIL5-resistant clones contained the transposon(s) in a carbamoyl transferase (KOP57525.1, e-value 9 × 10^−75^, 3/8), dTDP-glucose 4,6-dehydratase (KOP54966.1, e-value 3 × 10^−108^), dTDP-4-dehydrorhamnose reductase (KPY21988.1, e-value 6 × 10^−144^, 3/8), and/or a GDP-6-deoxy-D-lyxo-4-hexulose reductase (KPY25409.1, e-value 2 × 10^−8^, 1/8). In the case of KIL5, the knock-out of a DUF1294 domain-containing protein ALP22_101621 (RMU82419.1, e-value 5 × 10^−91^, 1/8) and a hypothetical membrane protein OX88_RS20865 (KOP53361.1, e-value 6 × 10^−64^, 7/8) showed full resistance to the phage. However, knocking-out the hypothetical membrane protein did not have an influence on the efficiency of KIL3b to infect Pspo (Appendix Figure A1). 

**Figure 1:**
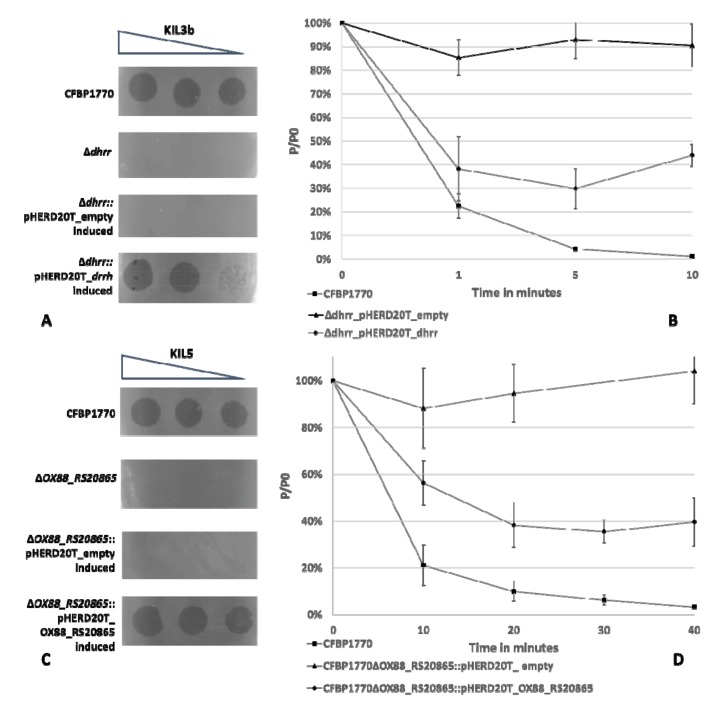
Complementation assay to test the effect of different genes on phage infection and adsorption. (**A**) Spot assay of KIL3b (10^8^, 10^7^ and 10^6^ PFU/mL) on a lawn of *Pseudomonas syringae* pv. *porri* (Pspo) CFBP 1770, CFBP 1770 Δ*dhrr*, CFBP 1770 Δ*dhrr*::pHERD20T_empty (induced) and CFBP 1770 Δ*dhrr*::pHERD20T_*dhrr* (induced), respectively. Expression of dTDP-4-dehydrorhamnose reductase (Dhrr) indeed restores phage infection. (**B**) Adsorption assay of KIL3b on CFBP 1770 (square), CFBP 1770 Δdhrr::pHERD20T_empty (induced) (triangle) and CFBP 1770 Δ*dhrr*::pHERD20T_*dhrr* (induced) (diamond) in which the percentage of non-adsorbed phages is plotted as a function of time. When Dhrr expression is restored, phages are able to adsorb to their host, confirming that this protein is involved in phage adsorption. (**C**) Spot assay of KIL5 (10^8^, 10^7^ and 10^6^ PFU/mL) on a lawn of CFBP 1770, CFBP 1770 Δ*OX88_RS20865*, CFBP 1770 Δ*OX88_RS20865*::pHERD20T_empty (induced) and CFBP 1770 Δ*OX88_RS20865*::pHERD20T_ *OX88_RS20865* (induced); also in this case, phage infection can be restored by introducing *OX88_RS20865*. (**D**) Adsorption assay of KIL5 on CFBP 1770 (square), CFBP 1770 Δ*OX88_RS20865*::pHERD20T_empty (induced) (triangle) and CFBP 1770 Δ*OX88_RS20865*::pHERD20T_*OX88_RS20865* (induced) (diamond). By overexpressing OX88_RS20865, phage adsorption to the host can partially be restored, indicating that this protein plays a role in the adsorption phase of KIL5.

To verify the relation of the dTDP-4-dehydrorhamnose reductase (Dhrr), involved in lipopolysaccharides (LPS) metabolism, and the membrane protein (OX88_RS20865) with an unknown function to phage infectivity of KIL3b and KIL5, respectively, the genes were cloned in a pHERD20T vector and transformed to the corresponding deletion mutants. A dilution series of the phage was spotted on a bacterial lawn containing the wild-type strains, the deletion mutants of the presumptive receptors, and complementation constructs for KIL3b and KIL5 (Figure 1A,C, respectively). This shows that indeed phage infection of both KIL3b and KIL5 can be restored by overexpression of the respective genes. However, the infectivity of KIL3b is not fully restored, as plaques show reduced size and appear turbid (Figure 1A). 

To prove these phenotypes are linked to phage adsorption efficiency, adsorption assays were performed (Figure 1B,D). KIL3b adsorbed to the wild-type host with an adsorption constant (*k*) of 4.47 × 10^−9^ mL/min, compared to a k-value of 7.63 × 10^−10^ mL/min for the complemented deletion mutant (after 10 min). In contrast, KIL5 had a constant of 1.51 × 10^−9^ mL/min and 5.53 × 10^−10^ mL/min when adsorbing to the wild type and the complemented deletion mutant in a timeframe of ten minutes, respectively. This difference in adsorption constant shows that KIL3b adsorbs more efficiently to its host compared to KIL5. Furthermore, it should be noted that the time frame for both phages was different. While KIL3b needed 10 minutes to adsorb to CFBP 1770, KIL5 required 40 minutes. In both cases, the adsorption of the phages to the host was restored when the bacterial mutant gene was complemented, albeit with a lower efficiency. Furthermore, a difference between the influence of the different genes could be observed. Restoring the function of Dhrr seemed to benefit the adsorption of KIL3b more compared to the effect that OX88_RS20865 had on the adsorption of KIL5. 

### 2.2. Phage-Resistant Pspo Mutants Display a Markedly Reduced Virulence

The effect of OX88_RS20865 on host virulence was evaluated. As such, CFBP 1770 Δ*OX88_RS20865* was injected in adult leek leaves and compared to wild-type CFBP 1770 and CFBP 1770 Δ*OX88_RS20865*::pHERD20T_ *OX88_RS20865*. In total, three leaves from five different plants were injected with bacterial suspension. After ten days, the lesion length was measured for leaves that had developed symptoms. Leaves for which the artificial infection had failed were removed from the dataset.

**Figure 2:**
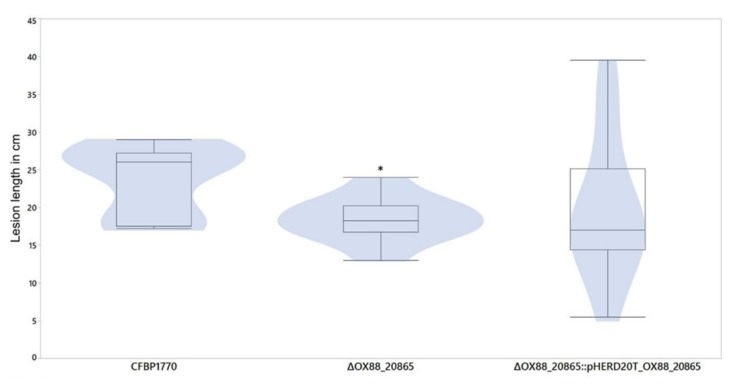
Virulence test on leek plants. The lesion length in cm of three different strains injected into the leaves. A significant difference (*) between CFBP 1770 and CFBP 1770 Δ*OX88_RS20865* can be observed using a nonparametric Wilcoxon test (*p*-value = 0.0148).

To compare the different groups, a Wilcoxon test (nonparametric) was applied as the measurements from the wild type were non-normally distributed (Shapiro–Wilk W test, *p*-value = 0.0171). As such, a significant difference was observed between the lesion length caused by wild-type CFBP 1770 and CFBP 1770 Δ*OX88_RS20865* (*p*-value = 0.0148) (Figure 2). Leaves injected with the complemented deletion mutant showed large variances in both length and symptom development.

### 2.3. Performance of the Phage Cocktail in Reducing Pspo Titers during Seed Priming

**Figure 3:**
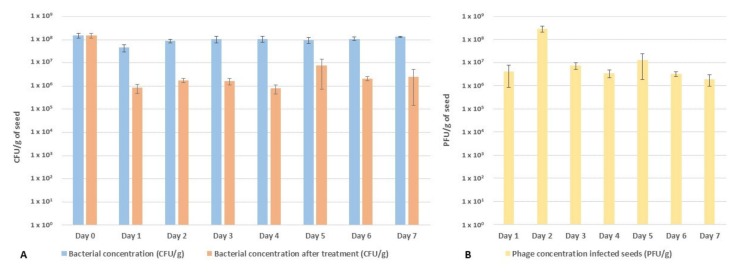
Monitoring bacterial and phage concentration during seed priming. (**A**) The bacterial titer (CFU/g seed) was followed as a function of time during a seven-day priming experiment in three independent repeats of non-treated and treated seeds in blue and orange, respectively. At day 0, the bacterial concentration was 10^8^ CFU/g. The bacterial titer dropped 100-fold in the infected seeds after treatment with phages at a final concentration of 10^9^ PFU/mL and remained stable over the course of the priming. (**B**) Phage titer (PFU/g seed) was followed over time in infected seeds. Twenty-four hours after treatment, the concentration of phage was 10^6^ PFU/g and increased after 48 h to 10^8^ PFU/g. After 72 h, the concentration dropped again to 10^7^ PFU/g and remained quite stable until day 7 (10^6^ PFU/g).

**Figure A2:**
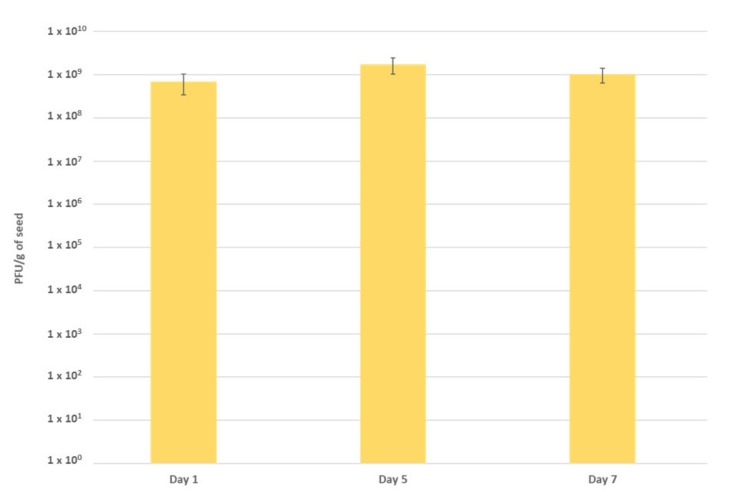
Phage titer (PFU/g) determined in noninfected seeds over a time period of seven days. The phage titer remains stable over the course of the seed priming (Day 7) at 10^9^ PFU/g seed.

The efficacy of a phage cocktail composed of KIL3b and KIL5 was tested in a seed priming experiment. Seeds artificially infected with CFBP 1770 (10^8^ CFU/g) were steeped in sterile mQ water to which phages were added (10^9^ PFU/mL) and incubated for seven days in three biological repeats. The bacterial concentration detectable in the seeds was monitored daily as well as the concentration of infectious phage particles (Figure 3A,B). It appeared that the concentration of Pspo in seeds remained quite steady around 10^8^ CFU/g over the duration of the experiment. When treated with phages, the concentration was a 100-fold reduction at Day 1, resulting in a final concentration of 10^6^ CFU/g (Figure 3A). Moreover, the concentration of phages determined on non-infected seeds remained quite steady at 10^9^ PFU/g over the course of the experiment (Day 1, Day 5, and Day 7) (Appendix Figure A2). However, the concentration of phages on Pspo-infected seeds dropped to 10^6^ PFU/g after 24 h and increased again at Day 2 to 10^8^ PFU/g. At Day 3, there was again a drop in the phage concentration to 10^7^ PFU/g, which slowly decreased over time to 10^6^ PFU/g (Figure 3B). 

### 2.4. Screening and Full Genome SNP Analysis of Phage-Resistant Pspo Strains

**Figure 4:**
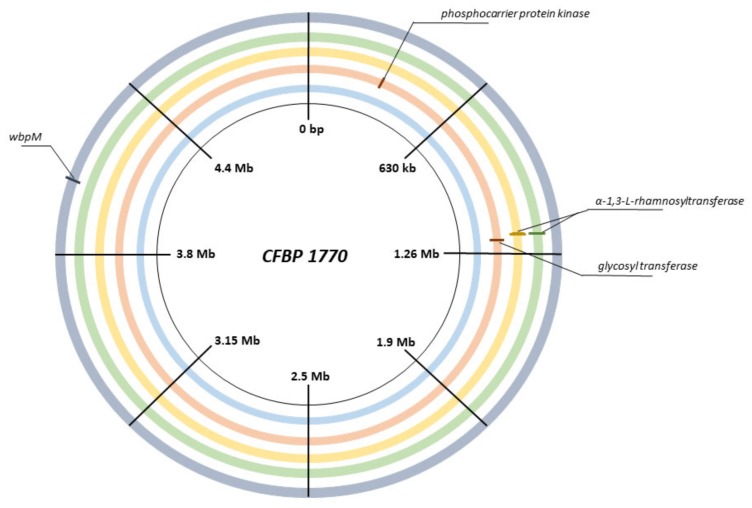
Genomic map of *Pseudomonas syringae* pv. *porri* CFBP 1770 and four phage-resistant mutants. The inner ring, in light blue, represents the reference genome. From the outside to the inside, Mutant 1 (grey), Mutant 2 (green), Mutant 3 (yellow), and Mutant 4 (orange) are displayed. The different SNPs are indicated on the represented genome map. Mutant 1 has a single SNP in *wbpM*, Mutant 2 and Mutant 3 both have a stop codon in α-1,3-L-rhamnosyltransferase, and Mutant 4 has a SNP in a phosphocarrier protein kinase and a deletion in a Family 2 glycosyltranferase.

Pspo isolates derived from the seed priming experiment were tested for their phage susceptibility by plating crushed seeds on phage agar plates. Seeds that were not treated with phages showed a total of 1 × 10^3^ (± 59) CFU/g phage-resistant Pspo clones, while the phage-treated seeds had a concentration of 4 × 10^6^ (± 8.2 × 10^5^) CFU/g of resistant mutants after seven days of incubation. This shows that the phage resistance frequency drastically increased in Pspo that were in contact with the phages. Four of these mutants that were resistant to both phages were analyzed using whole genome sequencing and compared to the reference strain through an SNP analysis. Only four mutations were identified as associated to the phage resistance. Mutant 1 had a point mutation in *wbpM*, a nucleotide sugar epimerase/dehydratase (Thr512Pro); Mutant 2 and Mutant 3 both gained a stop codon in an α-1,3-L-rhamnosyltransferase (Cys205*) and Mutant 4 acquired an SNP in a phosphocarrier protein kinase (Gly343Asp) and an out-of-frame deletion in a Family 2 glycosyl transferase (Figure 4). No additional deletions were found by screening the BAM pileups. 

## 3. Discussion

### 3.1. Elucidation of the Differences in Bacterial Receptors for Phages KIL3b and KIL5

As previously described, KIL3b and KIL5 are very similar at a genomic level, yet display differences in host range [13]. Interestingly, these differences are also represented at the level of the bacterial phylogeny, as the population of Pspo represented in the working collection can be categorized in two different clusters based on BOX-PCR data [10]. To successfully achieve infection, the phage needs to attach to its host, inject its genetic material, hijack the bacterial metabolism, replicate, and lyse the cell. At each level of this process, differences between bacterial strains can impact the outcome of phage infection [14]. In this research, we focused on the first step of infection, the adsorption phase, to see whether KIL3b and KIL5 use a different receptor or infection mechanism that contributes to the differences observed in host range. Furthermore, we evaluated the occurrence of phage-resistant mutants during a priming experiment. 

A transposon mutagenesis experiment revealed that knocking out dTDP-glucose-4,6-dehydratase, dTDP-4-dehydrorhamnose reductase, and/or GDP-6-deoxy-D-lyxo-4-hexulose reductase results in bacterial resistance to the phage cocktail of KIL3b and KIL5. These enzymes are all part of the nucleotide sugar metabolism. In Gram-negative bacteria, part of the products produced during this metabolism, especially rhamnose sugars in Pspo, are shuttled towards the production of lipopolysaccharides (LPS). Indeed, the Pspo O-antigen consists of L-rhamnose repeats [22]. LPS form a significant part of the outer membrane of Gram-negative bacteria and have previously been described to be recognized by certain phages as phage receptors [16]. To confirm the influence of the identified genes on KIL3b adsorption and more specifically the influence of a lack of functionalized L-rhamnose, CFBP 1770 Δ*dhrr* was further investigated. Phage infectivity was restored upon complementation with the wild-type gene, but not fully, as the plaque morphology was not fully restored. Anyway, it indicates that *dhrr* plays a crucial role in phage adsorption and thus we can conclude that both KIL3b and KIL5 recognize polysaccharides on the outer surface of Pspo. Sequencing analysis of natural phage-resistant Pspo mutants further confirmed this observation. Here, the natural mutants resistant to the cocktail showed mutations in *wbpM*, α-1,3-L-rhamnosyltransferase, and a Family 2 glycosyl tranferase, proteins involved in the production of LPS. Mutations that cause alterations in LPS molecules are generally associated with high pleiotropic costs, reducing fitness of the mutant [23]. Moreover, the relationship between LPS and virulence has previously been reported, such as the role of the membrane protein WbpM [24]. Hence, the link between losing a putative phage receptor, especially sugar moieties, and fitness and virulence of the pathogenic bacteria can further be highlighted, as has been described for multiple other pathosystems [25,26,27,28]. As such, one can postulate that bacteria that are resistant to these phages due to aberration in LPS pose lesser threats to their host plants. 

**Figure 5:**
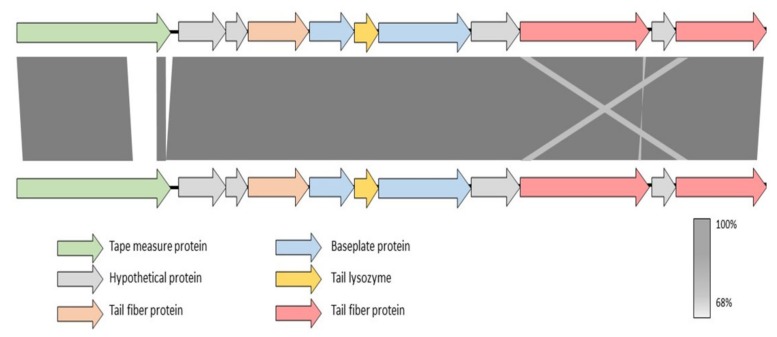
Tail fiber region of KIL3b (top) and KIL5 (bottom). The different genes are annotated as previously published [13]. Homology is based on nucleotide level (BLASTn). The tail fiber region of both phages is more or less identical except for the tape measure protein and a small translocation between the two major tail fiber proteins.

**Figure A3:**
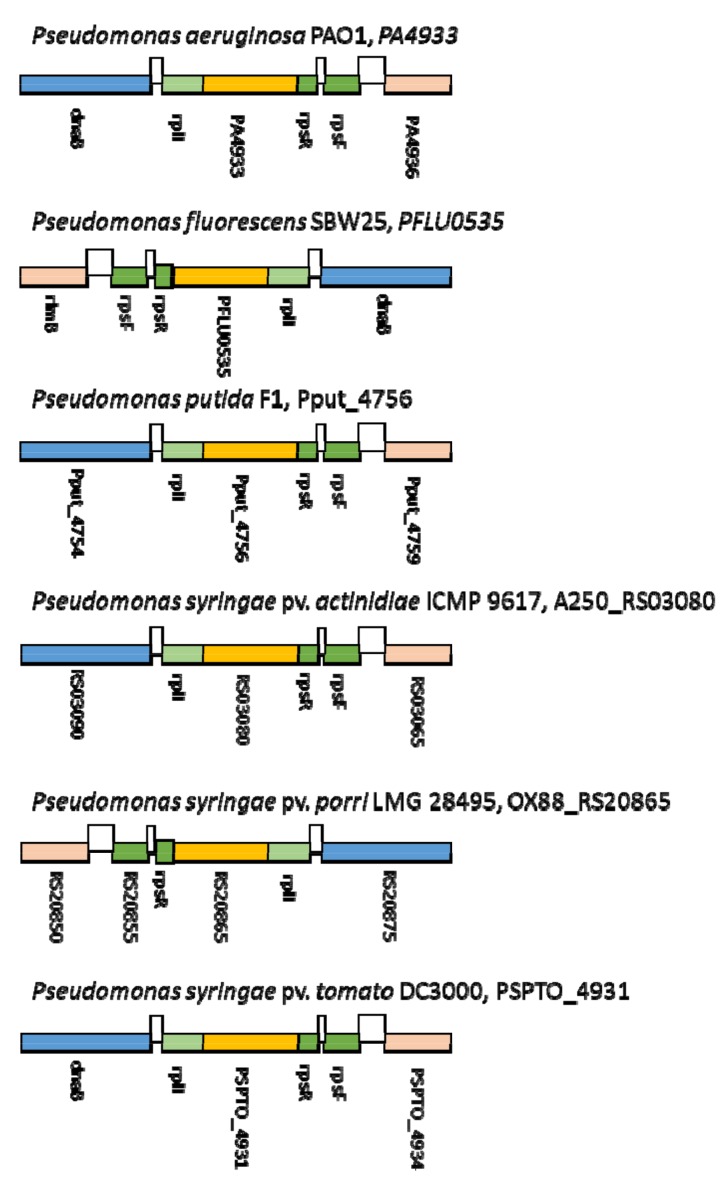
Genomic organization of the operon in which *OX88_RS20865* homologues are located in six different Pseudomonads: *Pseudomonas aeruginosa* PAO1, *Pseudomonas fluorescens* SBW25, *Pseudomonas putida* F1, *Pseudomonas syringae* pv. *actinidiae* ICMP 9617, *Pseudomonas syringae* pv. *porri* LMG 28495, and *Pseudomonas syringae* pv. *tomato* DC3000. The different functions of the genes are indicated with colors: blue—DNA helicase, light green—50s ribosomal subunit, yellow—OX88_RS20865 homologue, dark green—30s ribosomal subunit, pink—rRNA methyltransferase, white—intergenic region (adapted from www.pseudomonas.com).

In parallel, the receptor of KIL5 was evaluated as well. In contrast to KIL3b, knocking out *OX88_RS20865* strongly reduced KIL5 infection. After complementation, KIL5 plaque formation was identical to the wild type, although adsorption was only partially restored. The biological function of OX88_RS20865 remains to be elucidated, but the protein has transmembrane domains and a more detailed analysis shows that *OX88_RS20865* is widely distributed among members of the *Pseudomonas* genus. In *Pseudomonas aeruginosa* for example, *PA4933* is a homologue of *OX88_RS20865*. Here, the encoded protein is predicted to be part of the inner membrane. Interestingly, the genomic organization of this gene is conserved within the *Pseudomonas* genus and belongs to the same operon as *rplI, rpsR*, and *rps*, which are 50S and 30S ribosomal subunits (Appendix Figure A3). In terms of phage infectivity, inner membrane proteins such as OX88_RS20865 have been suggested to play a role in DNA injection [29]. It has been shown for example that *Escherichia coli* phage HK97 needs PtsG, an inner membrane glucose transporter, and FkpA, a periplasmatic chaperone, to interact with the phage tape measure protein (TMP) for DNA injection [29]. Evaluating the tail fiber region of KIL3b and KIL5 shows that they are almost identical, except for the C-terminal end of the TMP and a translocation between the genes coding for the major tail fibers (Figure 5). It has previously been reported that mutations in the TMP of phage λ heavily affects DNA injection [30,31]. Hence, we can hypothesize that the injection mechanism of KIL3b and KIL5 differ from each other, as there is no cross-resistance and KIL3b can still infect CFBP 1770Δ*OX88_RS20865*. This example illustrates that small differences in the tail fiber region are responsible for unique properties of highly similar phages and could even contribute to differences in host range. Using phages recognizing different receptors on the bacterial cell wall are considered to be more effective in controlling a bacterial infection [17,32]. The phages presented here appear to use a different infection mechanism and are characterized by different infection characteristics. As such, they are considered useful as biocontrol agents. 

Because little is known of the biological function of this inner membrane protein and as this protein is highly conserved in the *Pseudomonas* genus, the influence of OX88_RS20865 on the virulence of Pspo was evaluated by injecting CFBP 1770Δ*OX88_RS20865* directly into leek leaves. This bioassay demonstrates that there is a significant difference between the wild type and the deletion mutant, suggesting that OX88_RS20865 indeed contributes to Pspo virulence, by an unknown mechanism. 

### 3.2. Impact of the Bacterial Receptors on Phage-Based Biocontrol Assays

One of the limitations of phages is their applicability in field conditions, as the viruses are subjected to abiotic (and biotic) stresses (i.e., rain, wind, UV light, temperature) [2,33,34]. In some pathosystems, researchers try to overcome the limited stability of phages in these conditions by adding additives like skimmed milk and sugars which allow the phages to remain present on the aerial parts of the plants and increase their efficacy [33,35]. In the case of leek plants, the outbreak of the disease occurs through infected seeds or at plant nurseries [10,36]. A valid application strategy in this pathosystem would be to decontaminate seeds and thus prevent the outbreak of Pspo in the field. Hence, the performance of the phage cocktail and the development of resistance against the cocktail were evaluated in a seed priming experiment. Our artificially infected seed assay resulted in a 100-fold reduction of the bacterial titer. It should be noted that the in vitro artificial infection setting is far more stringent compared to the natural concentration of Pspo found on seeds (10^5^ CFU/g) observed in in vivo situations [36]. As such, the log2 reduction of Pspo observed here can be considered relevant and promising towards the application of phages in a biocontrol setting. Furthermore, it appears that the concentration of bacteria and phages remained quite stable over the course of the experiment, potentially illustrating an equilibrium between the two populations or even a coevolution dynamic [23]. Interestingly, not only did we observe a drop in the bacterial titer, but also a 100-fold reduction in the phage concentration compared to the control. It appeared that phage-resistant bacteria took over the population as the seed lots that were not treated with phage only contained 10^3^ CFU/g of resistant mutants, while the phage-treated seeds contained 10^6^ CFU/g of resistant Pspo mutants. Hence, we can argue that the 100-fold reduction of the bacterial titer is an underestimation of the total impact of the phage cocktail on the sensitive bacterial population. Another possible explanation for the observed fluxes in this assay could be due to the latency of the bacteria. One day after inoculation, the bacteria go into a non-active state and stop multiplying. As phages need bacteria with an active metabolism in order to successfully infect, their activity stops, and a status quo is reached. This hypothesis would also explain the observed drop in the phage titer. Phages in the priming solution still attach to their host, reducing the number of free phages in the priming solution. Sequencing four of these phage-resistant Pspo mutants showed that these clones accumulated mutations randomly in proteins responsible for LPS formation, further proving the importance of LPS as a phage receptor. It appears that the evolution pressure in Pspo is exerted at the level of the phage receptor. Similar results were shown in *P. aeruginosa* when infected with LPS-recognizing phages [37]. Extrapolating the fraction of naturally phage-resistant Pspo mutants in our tests, we can hypothesize that in naturally infected seeds, only a Pspo minority is resistant against the cocktail of the two phages. Randomly selected mutants from this population showed mutations in LPS producing genes, potentially mitigating the risk of infection by this phage-resistant fraction.

We can conclude that phages are indeed capable of actively infecting Pspo during a seed priming process. This demonstrates that the applicability of phages goes beyond spraying technologies and that tailor-made applications towards different pathosystems are relevant. The wide applicability of phages further highlights their potential as biocontrol agents within the framework of integrated pest management. However, as the occurrence of resistance is prominent and as other pathogens might take over, combining different biocontrol strategies will be important for future applications of phages. Integrated research is needed to give an answer to emerging crop diseases such as *P. syringae* pv. *porri* in leek plants. 

## 4. Materials and Methods 

### 4.1. Microbiological Manipulations

*P. syringae* pv. *porri* strain CFBP 1770 from the Collection Française de Bactéries Phytopathogènes (CFBP; Beaucouzé, France) was used as a reference strain. The bacteria were grown in lysogeny broth with reduced salt concentration (LB_ls_) (10 g/L Trypton, 5 g/L yeast extract and 0.5 g/L NaCl) at 25 °C or on LB_ls_ solid agar plates (1.5% bacteriological agar) or as soft agar overlays (0.7% agar). The two bacteriophages, KIL3b and KIL5, studied in this research belong to the *Flaumdravirus* genus (Myoviridae) and were previously isolated by our group [13,38]. Phages were amplified by infecting a liquid culture of Pspo CFBP 1770 at OD_600_ of 0.3 with an multiplicity of infection (MOI) of 0.01. After overnight incubation, the supernatant was filtered and PEG_8000_ precipitated to obtain a phage stock for downstream experiments. 

### 4.2. Tn5 Knock-Out Mutants of CFBP 1770 and Selection of Phage-Resistant Clones

A transposon knock-out library was generated using the EZ-Tn5<KAN-2>Tnp Transposome^TM^ kit (Epicentre (Lucigen), Middleton, WI, USA). Briefly, bacteria were grown to an OD_600_ of 0.5 and washed three times with 300 mM sucrose. Next, the cells were electroporated (12.5 kV/cm; 25 µF; 200 Ω) [39] and plated on selective medium (50 µg/mL kanamycin) and incubated for two days at 25 °C. Colonies were pooled and diluted to a final concentration of 10^8^ CFU/mL. To select for phage-resistant clones (KIL3b and KIL5, individually), the knock-out mutant library was infected with a multiplicity of infection (MOI) of 100 and incubated for two days at 25 °C. Growing colonies were picked, grown in liquid selective medium, and re-evaluated for their resistance by spotting 5x10^7^ PFU on top of a bacterial lawn containing the presumable phage-resistant clone. As such, clones that showed full resistance to either KIL3b or KIL5 were selected for further analysis. Cross-resistance of these fully resistant knock-out mutants to the other phage was tested in well plates by spotting a dilution series (10^6^ – 10^5^ – 10^4^ PFU/mL). DNA was extracted using the GeneJET Genomic DNA Purification Kit (ThermoFisher Scientific, Waltham, MA, USA). The location of the transposon inside the genome of the phage-resistant clones was determined by means of a thermal asymmetric interlaced PCR (TAIL PCR) as described elsewhere [40]. In short, the flanking regions of the transposon were amplified, Sanger sequenced, and analyzed using a tBlastx search [41]. 

### 4.3. Complementation of Phage Resistance and Phage Adsorption Assay

Interesting genes that could be involved in phage adsorption were cloned into a pHERD20T vector [42]. A stationary culture of the phage-resistant mutant of interest was centrifuged (13,000× *g*, 2 min) and washed three times with 300 mM sucrose. Next, the cells were electroporated (12.5 kV/cm; 25 µF; 200 Ω) with 5 ng of the pHERD20T construct and the transformants were grown on a selective, repressive medium containing 100 µM ampicillin and 2% glucose. To test the complementation of phage infectivity, a phage dilution series (5 µL of 10^6^ – 10^5^ – 10^4^ PFU/mL) was spotted on a bacterial lawn containing the complemented clone in the presence of 2% arabinose to induce the expression of the gene of interest. To test the effect on phage adsorption, adsorption assays were performed as described previously [43]. Briefly, bacteria were grown to an OD_600_ of 0.3 and infected with an MOI of 0.01. At specific time points, aliquots of 100 µL were taken and mixed with chloroform. These aliquots were titrated to determine the concentration of non-adsorbed phages. 

### 4.4. Virulence Assay in Leek Plants

The virulence of the deletion mutants and wild-type CFBP 1770 was compared in adult leek plants of cultivar Krypton (Nunhems) as described previously with minor modifications [13]. The veins of the leaf were damaged using a needle. A volume of 100 µL containing bacteria (10^8^ CFU/mL) was directly injected inside the leaf. In total, five plants and three leaves per plant were infected. Ten days after infection, the lesion length was measured. JMP Pro 14 software was used for the statistical analysis. 

### 4.5. Seed Bioassay 

Leek seeds (Rijkzwaan, The Netherlands) were surface sterilized and infected as described previously [44]. Briefly, leek seeds were sterilized using 5% hypochlorite, rinsed with sterile mQ water, and dried for one hour in a laminar flow cabinet. Next, the seeds were incubated in a bacterial suspension (CFBP 1770, at an OD_600_ of 0.1) for sixteen hours and dried for four hours under a laminar flow resulting in a final concentration of 10^8^ CFU/g seed. Sterile and infected seeds were steeped by shaking them at 16 °C for seven days in sterile mQ water. Phages were added at a final concentration of 10^9^ PFU/mL. Both phage and bacterial titers were monitored daily by homogenizing the seeds in 1 mL of PBS and titering the final suspensions. To quantify the bacterial concentration, dilutions were plated on selective *Pseudomonas* agar P (Difco Laboratories, Detroit, MI, USA) which allowed us to select Pspo based on colony morphology and the presence of pigments. 

### 4.6. Sequencing of Natural Phage-Resistant Clones of *Pseudomonas syringae* pv. *porri*

The Pspo mutants that survived the phage treatment in the seed infection test were further tested for phage resistance. Homogenized seeds in PBS were plated on solid agar plates on which a cocktail of 10^9^ KIL3b and KIL5 was spread. Thereafter, the 96 clones that grew on these plates were retested for phage susceptibility. Briefly, double agar overlays containing the 96 isolated clones were prepared in a 96-well plate. A phage cocktail containing KIL3b and KIL5 (10^10^ PFU/mL, 10 µL) was spotted on the bacterial lawn and incubated overnight at 25 °C. DNA from resistant clones was isolated using the Thermo Scientific genomic DNA isolation kit. Next, the genomic DNA was sequenced using our in-house MiniSeq Illumina NGS platform. The Nextera Flex DNA Library Kit was used for the library prep of the DNA. The average length (750 bp) of the DNA fragments was evaluated using an Agilent Bioanalyzer 2100 and a High Sensitivity Kit (Agilent Technologies, Santa Clara, CA, USA). The concentration was determined with Qubit (Thermo Fisher Scientific, USA) (600 pmol). Long reads of the reference genome were obtained using the MinION sequencer (Oxford Nanopore Technologies, Flowcell R9.4.1, using the Rapid Sequencing Barcoding Kit). Guppy (v3.1.5) was used for the base calling [45]. Illumina reads were trimmed with Trimmomatic (v0.36.5) and Nanopore reads were trimmed with Porechop (v0.2.4) [46,47]. A hybrid assembly of the reference genome was obtained with Unicycler (v0.4.8.0) [48] and evaluated using Bandage (v0.8.1) [49]. The final genome was annotated with using the PATRICbrc server (v3.6.2) [50]. The variants in the genomes of the mutants were determined with Snippy (v4.4.5+) [51]. The usegalaxy.eu platform was used for this bioinformatic analysis. 

## Appendix A

**Table A1:** Overview of the knock-out mutants and the location of the Tn5 transposon as identified during the TAIL-PCR that are potentially involved in phage infection.

Phage	KO Mutant	Protein	Accession Number	e-Value
KIL3b	CFBP 1770_R3a	dTDP-4-dehydrorhamnose reductase	KPY21988.1	6 × 10^−144^
	CFBP 1770_R3b	Carbamoyltransferase	KOP57525.1	9 × 10^−75^
	CFBP 1770_R3c	dTDP-4-dehydrorhamnose reductase	KOP54965.1	2 × 10^−98^
	CFBP 1770_R3d	Carbamoyltransferase	KOP57525.1	9 × 10^−75^
	CFBP 1770_R3e	Carbamoyltransferase	KOP57525.1	8 × 10^−75^
	CFBP 1770_R3f	dTDP-glucose 4,6-dehydratase	KOP54966.1	3 × 10^−108^
	CFBP 1770_R3g	GDP-6-deoxy-D-lyxo-4-hexulose reductase	KPY25409.1	2 × 10^−8^
KIL5	CFBP 1770_R5a	Hypothetical protein ALP22_101621	RMU82419.1	5 × 10^−91^
	CFBP 1770_R5b	Hypothetical membrane protein	KOP53361.1	4 × 10^−57^
	CFBP 1770_R5c	Hypothetical membrane protein	KOP53361.1	5 × 10^−50^
	CFBP 1770_R5d	Hypothetical membrane protein	KOP53361.1	6 × 10^−64^
